# Antibacterial and Antioxidant Activity of the Fruit of *Macaranga tanarius*, the Plant Origin of Taiwanese Green Propolis

**DOI:** 10.3390/antiox11071242

**Published:** 2022-06-24

**Authors:** Yi-Hsuan Chien, Yu-Hsiang Yu, Siou-Ru Ye, Yue-Wen Chen

**Affiliations:** Department of Biotechnology and Animal Science, National Ilan University, Yilan 26047, Taiwan; d0733003@niu.edu.tw (Y.-H.C.); yuyh@niu.edu.tw (Y.-H.Y.); ysru2008@gmail.com (S.-R.Y.)

**Keywords:** *Macaranga tanarius*, Taiwanese green propolis, propolin, antibacterial activity, antioxidant activity

## Abstract

Taiwanese green propolis (TGP) is widely used in traditional medicine and exerts a broad spectrum of biological activities, including those anti-inflammatory and anti-cancer in nature, resulting from an abundant level of functional propolins (prenylated flavanone) in the TGP. However, the plant origin of TGP has not been clarified. In this study, we collected the surface material of *Macaranga tanarius* fruit and comparatively analyzed the chemical composition, antibacterial activity, and antioxidant activity with TGP. The results revealed that there was no difference between the chemical composition of the glandular trichome extract of *M. tanarius* and those in propolis. Moreover, *M. tanarius* fruit extract was enriched in propolins (C, D, F, and G) and effectively inhibited the growth of Gram-positive strains. Propolins, TGP, and *M. tanarius* fruit extract showed powerful free radical-scavenging and ferrous-reducing activity. Collectively, we have confirmed the plant source of TGP is *M. tanarius*, and this plant has the enormous potential to be developed as a pharmaceutical plant due to the potent biological activities and the high amount of functional propolins.

## 1. Introduction

Propolis, a resinous substance collected by honeybees (*Apis mellifera*) from the leaves and buds of plants, is used to seal holes and cracks for making the hive more weathertight and to embalm dead insects or invaders. Propolis has been used as a folk medicine because of its broad spectrum of biological activities, such as those that are antiviral [1], antibacterial [2,3], antitumor [4], antioxidant [5], and anti-inflammatory [6].

The botanical source determines the chemical composition and the biological activity of propolis in the region [7]. The plant origin of numerous types of propolis has been identified. The Brazilian green propolis and European propolis are originated from the alecrim plant (*Baccharis dracunculifolia*) and poplar tree, respectively [8,9]. European propolis is rich in the phenolics that the poplar tree mainly contains, such as flavonoid aglycones, hydroxycinnamic acids and their esters [10]. The major components of Brazilian propolis are prenylated *p*-coumaric acid and diterpenic acids, which are the main compounds found in the *Baccharis* plants [11]. In the previous studies, we have found the Taiwanese green propolis (TGP) contained several prenylated flavanone derivatives and those are different from the above-mentioned propolis [12]. Taiwan is located in the east of Asia and possesses a subtropical climate. However, poplar trees and *Baccharis* plants cannot grow in these tropical and subtropical regions. Therefore, the plant origin of Taiwanese green propolis is expected to be vegetations that grow especially in the area.

Pacific propolis, also known as *Macaranga*-type propolis, predominantly derived from *Macaranga tanarius*, is mainly found in Indonesia, Hawaii, and the Okinawa prefecture of Japan [13]. Kumazawa et al. (2008) [14] demonstrated that the surface resinous material (glandular trichome) of *M. tanarius* fruit is the plant source of the Okinawa propolis (OP) by observing the behavior of honeybees in combination with comparative chemical analysis of propolis and plant material. Several studies have reported that the major compound of OP is prenylated flavanone and has a high degree of similarity with TGP [15,16]. Moreover, it has been documented that Hawaii propolis (HP) contains the nine prenylated flavonoids that have been isolated from OP [17]. Due to the high similarity of the major compounds and close geographical location, TGP has been categorized into *Macaranga*-type propolis. However, to our knowledge, no studies have really confirmed the plant origin of the TGP.

**Figure 1:**
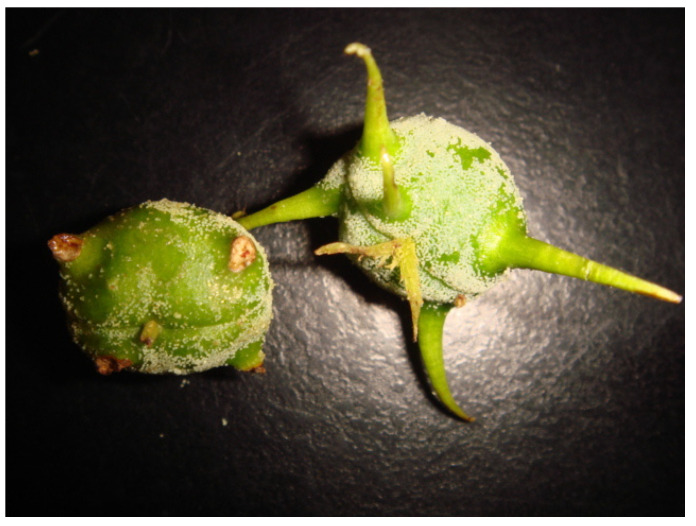
New (**right**) and mature (**left**) fruit of *M*. *tanarius*.

Currently, there are ten prenylated flavanone derivatives, and propolins A-J have been isolated from Taiwanese green propolis (TGP) and characterized [5,12,18,19]. TGP has been reported to have a broad spectrum of biological activities, including those that are anticancer [19], anti-inflammatory [6,20] and antioxidant [5]. If we compare TGP with OP, propolin C, D, F, G, and H in TGP are identical to nymphaeol A, nymphaeol B, isonymphaeol B, nymphaeol C, and 3′-geranyl-naringenin in OP, respectively [21]. *M. tanarius* is widely distributed in tropical areas of Asia, including the south of Japan, the Philippines, Malaysia, India, Thailand, China, and Taiwan [22]. Therefore, we hypothesized that *M. tanarius* is principally the botanical source of TGP. The objective of this work is to confirm the plant origin of TGP by comparatively analyzing the chemical composition, antioxidant activity, and antibacterial activity of the material of *M. tararius* fruit with TGP. It has been reported that the OP is originated from the surface white resinous material of *M. tanarius* fruit [14]. *M. tanarius* fruits could be separated into new fruit and mature fruit according to the time length following production. The soft thorns of new fruit are complete and evenly covered with white resinous materials, while the surface of mature fruit is scratched (Figure 1). In the present study, we collected the surface material of *M. tanarius* new and mature fruits to confirm the stability of the chemical components in the plant source of TGP by comparative chemical analysis and antioxidant activity.

## 2. Materials and Methods

### 2.1. Sampling of M. tanarius Fruit and Propolis

*M. tanarius* was collected from Yilan, Taiwan in June 2015. *M. tanarius* plants were separated into leaf, flower, stalk, and fruit. The fruits were further separated into seed and pericarp. Each part was air-dried for 3 days and ground using a grinder. For glandular trichome collection, *M. tanarius* fruits were divided into new fruits or mature fruits based on the appearances shown in Figure 1. The surface nonfood material (glandular trichome) of *M. tanarius* fruits was scraped by a steel spatula. TGP was provided by Yong Shyang Honey Enterprise Co., Ltd., Changhua, Taiwan, and it was initially collected from beehives located in different regions in Taiwan from May to July 2015 using propolis collectors. The source of TGP used in this experiment is the same as in the previous study [2]. The ground propolis and each part of *M. tanarius* plant were extracted with methanol at a ratio of 1:10 (*w*/*v*) by shaking (250 rpm) at 25 °C for 48 h. The extracts were then filtered through a filter paper and reconstituted to their original volume with methanol.

### 2.2. High-Performance Liquid Chromatography Analysis

The HPLC analysis was performed with an Agilent 1200 HPLC system (Santa Clara, CA, USA) fitted with a programmable UV detector, equipped with a reverse phase RP-18 column (ZORBAX SB-C18, 4.6 × 250 mm: Agilent, Santa Clara, CA, USA). The mobile phase consisted of water: methanol (88.8:11.2, *v*/*v*). The flow rate was 1 mL/min. The elution of extracts was monitored at 280 nm by UV detector. The standards of propolins (C, D, F, and G) were isolated from TGP by HPLC. Standards of propolins (C, D, F, and G) were analyzed and the concentration of propolins in the sample was determined by the standard curve based on the peak area for each propolin.

### 2.3. Measuring the Antioxidant Power

The TGP extract, *M. tanarius* fruit extracts, and propolins (C, D, F, and G) standard were concentrated by vacuum evaporation, then dissolved in methanol and serially diluted (concentration range from 5.0 to 160.0 μg/mL). For DPPH scavenging assay, the free radical-scavenging capacities of extract samples were measured spectrophotometrically following mixing 100 μL 500 μM DPPH methanolic solution and 100 μL samples. After 1 h incubation at room temperature in the dark, the absorbance was recorded at 517 nm. Methanol was used as the blank control. Caffeic acid phenethyl ester was used as a positive control. The degradation of DPPH was evaluated by comparison with a blank control. The capability of scavenging DPPH radicals was then calculated by the following equation: Scavenging effect (%) = [1 − (*A*_517_ of sample/*A*_517_ of control)] × 100. IC_50_ (half maximal inhibitory concentration) value denotes the concentration of sample required to scavenge 50% DPPH radicals. The software (CalcuSyn, Biosoft, St. Louis, MO, USA) was used to calculate the concentration (IC_50_) required to remove 50% DPPH radicals.

For ABTS radical cation scavenging assay, ABTS^•+^ radicals were generated by mixing ABTS aqueous solution (7 mM) with 2.45 mM potassium persulfate (final concentration) in the dark for 12–16 h at room temperature. The solution was diluted with ethanol to the 0.70 ± 0.05 at 734 nm. The samples (10 μL) were added to 190 μL diluted ABTS^•+^ solution, and the absorbance was measured at 734 nm after 5 min. Ethanol was used as the blank control. The capability of scavenging ABTS^•+^ radicals of samples were presented as IC_50_.

For FRAP assay, the ferric-ion-reducing activity of samples was measured using commercial Ferric Reducing Antioxidant Power (FRAP) Assay Kit (Sigma-Aldrich, St. Louis, MO, USA) according to the manufacturers’ instructions. The FRAP value was expressed as mM Ferrous equivalents of samples (g).

### 2.4. Test Organisms

All bacterial strains were purchased from the Food Industry Research and Development Institute (Hsinchu, Taiwan). *Staphylococcus aureus* (BCRC 10780, BCRC 10781 and BCRC 10451) and *Bacillus cereus* (CCRC 10603) were cultured in tryptic soy broth (TSB, Difco, Sparks, MD, USA). *Bacillus subtilis* (BCRC 10255), *Escherichia coli* (BCRC 10675) and *Pseudomonas aeruginosa* (BCRC 10944) were cultured in nutrient broth (NB, Difco Laboratories, Detroit, MI, USA). After successfully subculturing test organisms twice, the activated culture was inoculated into culture media to achieve an assay concentration of 1~5 × 10^5^ CFU/mL.

### 2.5. Minimum Inhibitory Concentration and Minimum Bactericidal Concentration

The micro dilution method in 96-well microtiter plates was used to study the minimum inhibitory concentration (MIC) of TGP and *M. tanarius* fruit extracts. The dry extracts were dissolved in dimethyl sulfoxide (DMSO, Sigma, St. Louis, MO, USA) and serially diluted (concentration range from 0.625 to 640.0 µg/mL). Each test well contained 10 µL sample solutions and 90 µL culture broth and further inoculated with 100 µL bacterial suspension (1~5 × 10^6^ CFU/mL). Sterility control and growth control were prepared. The MIC value of the extract was defined as the lowest concentration that completely prevented the growth of each microorganism after 48 h of incubation at 37 °C by analyzing the turbidity of bacterial growth at 595 nm. For the determination of MBC, 10 µL of liquid culture from each well that showed no apparent growth were taken and sub-cultured on fresh agar plates then incubated at 37 °C for 24 h. The MBC value was read as the least concentration exhibiting no visible growth on plates. All experiments were performed in triplicate.

### 2.6. Statistical Analysis

Data are expressed as Mean ± SD and were tested for statistical significance by one-way ANOVA with least significant difference (LSD) post hoc tests when multiple groups were compared and Student *t*-tests when the two groups were compared. The *p* value less than 0.05 was considered statistically significant. Data were analyzed using SAS (SAS Institute, Cary, NC, USA).

## 3. Results

### 3.1. Analysis of the Surface Material Extract of M. tanarius Fruit

**Figure 2:**
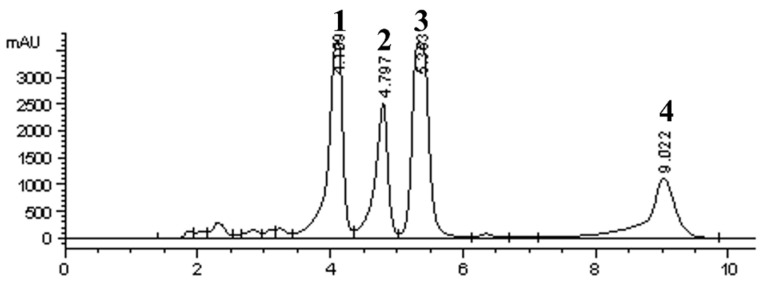
HPLC profile of the surface material of *M. tanarius* new fruit extract. Three experiments were carried out, and one representative result is shown. Peaks: 1: propolin D; 2: propolin F; 3: propolin C; 4: propolin G.

The dry matter yield and the level of propolins of each part of the *M. tanarius* extracts are shown in Table 1. The glandular trichome extract of *M. tanarius* new fruit was found to have the highest dry matter yield (73.96 ± 0.13%). The maximum yield of total propolins (C, D, F, and G) and the individual level of propolins were also observed in the glandular trichome extract of *M. tanarius* new fruit. In the leaf, flower, stalk, pericarp, and seed, both dry matter yield and the propolin content were low. The HPLC profile of new fruit extract was shown in Figure 2. Four peaks were assigned by comparing the retention times (RT) and UV spectra of HPLC chromatograms (280 nm) of the propolin standards (C, D, F, and G) we have previously reported [2]. Peaks 1, 2, 3, and 4 are equal to propolin D, propolin F, propolin C, and propolin G, respectively [2]. The HPLC profile of new fruit extract and TGP exhibited high consistency.

### 3.2. Antibacterial Activity of Extracts

The average MIC and MBC of TGP methanol extracts for Gram-positive strains was 10–40 μg/mL (Table 2). The *M. tanarius* fruit extracts have lower both MIC and MBC against Gram-positive microbes than TGP methanol extract with MIC ranging from 1.25 μg/mL to 10 μg/mL. The new fruit and mature fruit extracts showed similar antibacterial activity against Gram-positive bacteria, but the new fruit extract exerted a more potent bactericidal effect against *S. aureus* (BCRC 10451) and *B. subtilis*. However, none of the three extracts was able to inhibit the growth of Gram-negative strains, including *E. coli* and *P. aeruginosa*. These results suggested that both TGP extract and *M. tanarius* fruit extract were able to inhibit Gram-positive bacteria growth but had no antibacterial effect on Gram-negative bacteria.

### 3.3. Antioxidant Activity of the Extracts and Propolins

In the previous study, we reported that TGP has strong DPPH radical scavenging activity [23]. As shown in Table 3, the *M. tanarius* fruit extracts exhibited stronger free radical-scavenging activity with lower IC_50_ than TGP extract. There were no significant differences found in free-radical scavenging activity between new fruit and mature fruit.

The results of ABTS and FRAP assay were shown in Table 4. The *M. tanarius* new fruit extract scavenged ABTS radicals more efficiently than TGP extract, and there were no significant differences found in ferric-reducing antioxidant power. We also evaluated the antioxidant activity of propolins C, D, F, and G. The results were shown in Table 5. Caffeic acid phenethyl ester (CAPE) is one of the main active ingredients of poplar-type propolis. It has been reported that CAPE exerted excellent antioxidant activity [24]. As shown in Table 5, CAPE had the strongest free radical-scavenging activity with the lowest IC_50_ (µM) than all individual propolins in DPPH and showed powerful ferric-reducing power with the highest amount of ferrous, but ABTS radical-scavenging power was inferior to propolin C and G. Among the propolins, propolin C scavenged free radical and reduced ferric more efficiently than other propolins. Only propolin C had lower IC_50_ (µg/mL) than the *M. tanarius* fruit extracts and TGP extract in the DPPH assay. Propolin C also showed significantly lower IC_50_ (µg/mL) in the ABTS assay, and higher ferrous equivalent than the *M. tanarius* new fruit extract and TGP extract. These results suggest that propolins C, D and G may contribute to the antioxidant capability of *M. tanarius* fruit extracts and TGP.

## 4. Discussion

In the tropical region, *M. tanarius* has been used in folk medicine. In Vietnam, this plant has been used in traditional medicine for treating furuncles [25]. In Malaysia and Thailand, a decoction of the root of *M. tanarius* is used as an antipyretic and an antitussive. The dried root is used as an emetic agent, whereas the fresh leaves are used to cover wounds to prevent inflammation. In addition, the young shoots are eaten as a vegetable source in Thailand [26]. In Taiwan, the dried leaves of *M. tanarius* is used in herbal tea [27]. *M. tanarius* is widely distributed in the plains and low-altitude area of Taiwan and its time of fructification is consistent with the production period of TGP [23]. We previously have found a high amount of propolins C and D in methanolic extracts of buds and young leaves of the Euphorbiaceae plant in Taiwan [23]. In the present study, we collected the surface material of *M. tanarius* fruits to conduct comparative chemical analysis with TGP because it has been documented that the honeybees collect the glandular trichome of *M. tanarius* fruit and use it to produce propolis in Okinawa, Japan [14]. Several prenylated flavanones isolated from the glandular trichome of *M. tanarius* were also found in the leaf of *M. tanarius* [28,29,30]. Moreover, Kumazawa et al. (2014) [16] quantitatively analyzed the prenylflavonoids in various parts of *M. tanarius* and demonstrated that propolins (C, D, F, and G) were also present in not only leaf and glandular trichome but also petiole, leaflet, flower, seed, and pericarp. These results are consistent with our finding that different parts of the *M. tanarius* plant also contain propolins. Currently, we have confirmed that the plant source of TGP is *M. tanarius* via comparative chemical analysis. Chen et al. (2008) [23] confirmed that Taiwanese propolis (TP) can be categorized into three types based on color and season: TW-I (green, May–July), TW-II (brownish green, August–October) and TW-III (dark brown, October–December). They concluded that the season is a key factor in determining the level of propolins in TP. Collectively, the color differences and propolins content probably arose from natural seasonal changes of the botanical origins. The early summer is the fruiting season of *M. tanarius*, which is consistent with the production period of TGP (TW-I). It means non-green Taiwanese propolis may come from the different parts of *M. tanarius* or other plants. Further study is needed to clarify. We previously found that the total amount of propolins (C, D, F, and G) in a methanol extract of propolis was 333.40 ± 2.15 mg/g [2]. However, this is much lower than the yield of propolins (C, D, F, and G) of *M. tanarius* new fruit (626.70 ± 0.37 mg/g) and mature fruit (567.80 ± 0.36 mg/g). Moreover, the proportion of propolin (C, D, F, and G) content of *M. tanarius* glandular trichome extract is up to 84%. In contrast, those in TGP extract contain only 56%. During the process of honeybees collecting the propolis, their beeswax and other various substances may be mixed into the propolis, causing the collections to be diluted. The glandular trichome of *M. tanarius* fruit extract, containing purely the high amount of propolins, is worth developing into health supplements by abundantly extracting the propolins. 

Here, we observed that the glandular trichome of *M. tanarius* fruit containing propolins exhibited powerful antibacterial activity against Gram-positive strains instead of Gram-negative strains. The methanolic leaf extract of *M. tanarius* containing propolins (C, D, F, G, and H) is able to inhibit the growth of the Gram-positive strains including *B. cereus*, *S. aureus*, and *Micrococcus luteus*, but no activity was observed for the Gram-negative species [16,31]. The Solomon propolis containing propolins (C, D, G, and H) exhibited antibacterial activity against MRSA with MIC values in the range of 64–128 μg/mL [32]. In the previous study, we have confirmed that propolin C exhibited the highest antibacterial activity against Gram-positive strains, while none of the propolins had antibacterial activity against Gram-negative strains [2]. These results suggest that *M. tanarius* fruit extract has antibacterial capacities attributed to propolins.

In this study, we observed that the *M. tanarius* fruit extract containing propolins exhibited potent free radical-scavenging activity. This is the first report showing the DPPH and ABTS radical-scavenging activity of individual propolin. Matsunami et al. (2006) [33] reported that the leaf of *M. tanarius* possesses potent DPPH radical-scavenging activity. Previous study suggested that propolins C, D, and F may contribute to the free radical-scavenging capability of TGP [23]. These results are partially consistent with our finding that propolins C, D, and G contribute to the free radical-scavenging capability of TGP and propolins presenting together in TGP may give the synergistic effect.

## 5. Conclusions

In conclusion, this is the first report with clear evidence demonstrating that the plant origin of TGP is *M. tanarius* by comparative chemical and biological analysis. Further, we have clarified that propolins have free radical-scavenging activity and contribute to the antibacterial and antioxidant activity of *M. tanarius* extract and TGP. *M. tanarius* has a potential to be developed into a functional pharmaceutical plant, especially the glandular trichome of *M. tanarius* new fruit due to its potent biological activities and the high amount of functional propolins.

## Figures and Tables

**Table 1:** Dry matter yield (%) and propolin content (mg/g) in different parts of *M. tanarius*.

Plant Part	Yield (%)	Propolin C (mg/g)	Propolin D (mg/g)	Propolin F (mg/g)	Propolin G (mg/g)	Propolin C + D + F + G (mg/g)	
Leaf	19.75 ± 0.96 *^,d^	1.60 ± 0.09 ^g^	3.65 ± 0.07 ^e^	1.52 ± 0.04 ^e^	16.38 ± 0.58 ^d^	23.14 ± 0.74 ^d^	
Flower	10.00 ± 0.82 ^e^	2.31 ± 0.12 ^f^	1.97 ± 0.09 ^g^	0.83 ± 0.04 ^g^	4.10 ± 0.18 ^e^	9.21 ± 0.42 ^e^	
Stalk	6.75 ± 0.50 ^g^	0.26 ± 0.01 ^h^	0.32 ± 0.05 ^h^	0.47 ± 0.10 ^h^	0.44 ± 0.10 ^g^	1.49 ± 0.25 ^f^	
Pericarp	8.25 ± 0.50 ^f^	8.96 ± 0.30 ^d^	6.37 ± 0.27 ^d^	2.95 ± 0.07 ^d^	4.04 ± 0.20 ^e^	22.31 ± 0.83 ^d^	
Seed	8.00 ± 0.00 ^f^	3.33 ± 0.09 ^e^	2.87 ± 0.06 ^f^	1.33 ± 0.03 ^f^	1.77 ± 0.06 ^f^	9.30 ± 0.24 ^e^	
Glandular trichome of new fruit	73.96 ± 0.13 ^a^	223.30 ± 0.16 ^a^	142.10 ± 0.12 ^a^	107.2 ± 0.08 ^a^	154.10 ± 0.18 ^a^	626.70 ± 0.37 ^a^	
Glandular trichome of mature fruit	67.90 ± 0.30 ^b^	217.30 ± 0.12 ^b^	119.50 ± 0.08 ^b^	81.9 ± 0.05 ^b^	149.00 ± 0.15 ^b^	567.80 ± 0.36 ^b^	
TGP	59.75 ± 1.26 ^c^	133.10 ± 0.88 ^c^	69.30 ± 0.40 ^c^	39.30 ± 0.27 ^c^	91.80 ± 0.66 ^c^	333.40 ± 2.15 ^c^	

* Data are presented as means ± SD (*n* = 3). Statistically significant differences are indicated by different lowercase letters (*p* < 0.05, one-way ANOVA with LSD post hoc test).

**Table 2:** MIC and MBC (µg/mL) of TGP extract and the surface material of *M. tanarius* fruit.

	New Fruit		Mature Fruit		TGP	
Bacteria	MIC	MBC	MIC	MBC	MIC	MBC
*S. aureus* (BCRC 10780)	5	10	5	10	20	20
*S. aureus* (BCRC 10781)	5	10	5	10	20	20
*S. aureus* (BCRC 10451)	10	10	10	20	20	40
*B. subtilis*	1.25	2.5	1.25	5	10	20
*B. cereus*	1.25	2.5	1.25	2.5	20	20
*E. coli*	>640	>640	>640	>640	>640	>640
*P. aeruginosa*	>640	>640	>640	>640	>640	>640

Values are expressed as means of triplicate analyses for each sample (*n* = 3).

**Table 3:** IC_50_ of TGP and the surface material extract of *M. tanarius* fruits in scavenging DPPH radicals.

Source	IC_50_ (µg/mL)
New fruit	14.47 ± 0.38 *^,b^
Mature fruit	14.48 ± 0.44 ^b^
TGP	17.63 ± 0.90 ^a^

* Data are presented as means ± SD (*n* = 3). Statistically significant differences are indicated by different lowercase letters (*p* < 0.05, one-way ANOVA with LSD post hoc test).

**Table 4:** Antioxidant activity of TGP and the surface material extract of *M. tanarius* new fruit in scavenging ABTS radicals and reducing ferric (Fe^3+^) ion.

	ABTS	FRAP
Source	IC_50_ (µg/mL)	mmol Fe^2+^/g
New fruit	21.24 ± 1.31 *	5.52 ± 0.55
TGP	27.6 ± 0.73	4.59 ± 1.83

Data are presented as means ± SD (*n* = 3). * *p* < 0.05.

**Table 5:** Antioxidant activity of propolins.

	DPPH		ABTS		FRAP
Propolin	IC_50_ (µg/mL)	IC_50_ (µM)	IC_50_ (µg/mL)	IC_50_ (µM)	mmol Fe^2+^/g
C	12.98 ± 0.38 *^,d^	30.61 ± 0.90 ^c^	18.21 ± 0.23 ^c^	42.95 ± 0.56 ^c^	9.65 ± 1.52 ^b^
D	17.56 ± 0.58 ^c^	41.42 ± 1.37 ^b^	23.96 ± 1.62 ^b^	56.51 ± 4.62 ^b^	3.18 ± 0.65 ^d^
F	22.29 ± 0.36 ^a^	52.57 ± 0.85 ^a^	33.91 ± 1.41 ^a^	68.92 ± 3.33 ^a^	3.17 ± 0.72 ^d^
G	20.79 ± 0.27 ^b^	42.26 ± 0.55 ^b^	20.35 ± 0.89 ^b^	41.36 ± 1.81 ^c^	5.39 ± 0.62 ^c^
CAPE	7.99 ± 0.24 ^e^	28.10 ± 0.84 ^d^	16.72 ± 0.16 ^c^	58.81 ± 2.15 ^b^	12.74 ± 1.28 ^a^

CAPE, Caffeic acid phenethyl ester. * Data are presented as means ± SD (*n* = 3). Statistically significant differences are indicated by different lowercase letters (*p* < 0.05, one-way ANOVA with LSD post hoc test).

## Data Availability

The data are contained within the article.

## References

[1] Kujumgiev A., Tsvetkova I., Serkedjieva Y., Bankova V., Christov R., Popov S. (1999). Antibacterial, antifungal and antiviral activity of propolis from different geographic origins. J. Ethnopharmacol..

[2] Chen Y.W., Ye S.R., Chen. C.T., Yu Y.H. (2018). Antibacterial activity of propolins from Taiwanese green propolis. J. Food Drug Anal..

[3] Chen C.T., Chien Y.H., Yu Y.H., Chen Y.W. (2019). Extraction and analysis of Taiwanese green propolis. J. Vis. Exp..

[4] Bassani-Silva S., Sforcin J.M., Amaral A.S., Gaspar L.F.J., Rocha N.S. (2007). Propolis effect in vitro on canine transmissible venereal tumor cells. Rev. Port. Cienc. Vet..

[5] Chen C.N., Weng M.S., Wu C.L., Lin J.K. (2004). Comparison of radical scavenging activity, cytotoxic effects and apoptosis induction in human melanoma cells by Taiwanese propolis from different sources. Evid.-Based Complementary Altern. Med..

[6] Hsieh C.Y., Li L.H., Rao Y.K., Ju T.C., Nai Y.S., Chen Y.W., Hua K.F. (2019). Mechanistic insight into the attenuation of gouty inflammation by Taiwanese green propolis via inhibition of the NLRP3 inflammasome. J. Cell. Physiol..

[7] Bankova V., Bertelli D., Borba R., Conti B.J., da Silva Cunha I.B., Danert C., Eberlin M.N., Falcão S.I., Isla M.I., Moreno M.I.N. (2016). Standard methods for *Apis mellifera* propolis research. J. Apic. Res..

[8] Bankova V., de Castro S.L., Marcucci M.C. (2000). Propolis: Recent advances in chemistry and plant origin. Apidologie.

[9] Kumazawa S., Yoneda M., Shibata I., Kanaeda J., Hamasaka T., Nakayama T. (2003). Direct evidence for the plant origin of Brazilian propolis by the observation of honeybee behavior and phytochemical analysis. Chem. Pharm. Bull..

[10] Bankova V., Popova M., Bogdanov S., Sabatini A.G. (2002). Chemical composition of European propolis: Expected and unexpected results. Z. Naturforsch..

[11] Park Y.K., Alencar S.M., Aguiar C.L. (2002). Botanical origin and chemical composition of Brazilian propolis. J. Agric. Food Chem..

[12] Huang W.J., Huang C.H., Wu C.L., Lin J.K., Chen Y.W., Lin C.L., Chuang S.E., Huang C.Y., Chen C.N. (2007). Propolin G, a prenylflavanone, isolated from Taiwanese propolis, induces caspase-dependent apoptosis in brain cancer cells. J. Agric. Food Chem..

[13] Sforcin J.M., Bankova V. (2011). Propolis: Is there a potential for the development of new drugs?. J. Ethnopharmacol..

[14] Kumazawa S., Nakamura J., Murase M., Miyagawa M., Ahn M.R., Fukumoto S. (2008). Plant origin of Okinawan propolis: Honeybee behavior observation and phytochemical analysis. Sci. Nat..

[15] Kumazawa S., Goto H., Hamasaka T., Fukumoto S., Fujimoto T., Nakayama T. (2004). A new prenylated flavonoid from propolis collected in Okinawa, Japan. Biosci. Biotechnol. Biochem..

[16] Kumazawa S., Murase M., Momose N., Fukumoto S. (2014). Analysis of antioxidant prenylflavonoids in different parts of *Macaranga tanarius*, the plant origin of Okinawan propolis. Asian Pac. J. Trop. Med..

[17] Inui S., Hosoya T., Kumazawa S. (2014). Hawaiian propolis: Comparative analysis and botanical origin. Nat. Prod. Commun..

[18] Chen C.N., Wu C.L., Shy H.S., Lin J.K. (2003). Cytotoxic prenylflavanones from Taiwanese propolis. J. Nat. Prod..

[19] Chen C.N., Wu C.L., Lin J.K. (2007). Apoptosis of human melanoma cells induced by the novel compounds propolin A and propolin B from Taiwanese propolis. Cancer Lett..

[20] Hsiao F.S.H., Artdita C.A., Hua K.F., Tsai C.J., Chien Y.H., Chen Y.W., Cheng Y.H., Yu Y.H. (2022). Optimization of emulsification conditions on ethanol extract of Taiwanese green propolis using polysorbate and its immunomodulatory effects in broilers. Antioxidants.

[21] Shahinozzaman M., Obanda D.N., Tawata S. (2021). Chemical composition and pharmacological properties of Macaranga-type Pacific propolis: A review. Phytother.

[22] Hatusima S. (1975). Flora of the Ryukyus. Added and Corrected.

[23] Chen Y.W., Wu S.W., Ho K.K., Lin S.B., Huang C.Y., Chen C.N. (2008). Characterization of Taiwanese propolis collected from different locations and seasons. J. Sci. Food Agric..

[24] Ozer M.K., Parlakpinar H., Acet A. (2004). Reduction of ischemia-reperfusion induced myocardial infarct size in rats by caffeic acid phenethyl ester (CAPE). Clin. Biochem..

[25] Chi V.V. (1997). Vietnamese Medical Plant Dictionary.

[26] Chulabhorn M., Prawat H., Prachyawarakorn V., Ruchirawat S. (2002). Investigation of some bioactive Thai medicinal plants. Phytochem. Rev..

[27] Grosvenor P.W., Gothard P.K., McWilliam N.C., Supriono A., Gray D.O. (1995). Medicinal plants from Riau Province, Sumatra, Indonesia. J. Ethnopharmacol..

[28] Phommart S., Sutthivaiyakit P., Chimnoi N., Ruchirawat S., Sutthivaiyakit S. (2005). Constituents of the leaves of *Macaranga tanarius*. J. Nat. Prod..

[29] Guhling O., Kinzler C., Dreyer M., Bringmann G., Jetter R. (2005). Surface composition of myrmecophilic plants: Cuticular wax and glandular trichomes on leaves of *Macaranga tanarius*. J. Chem. Ecol..

[30] Kawakami S., Harinantenaina L., Matsunami K., Otsuka H., Shinzato T., Takeda Y. (2008). Macaflavanones A-G, prenylated flavanones from the leaves of *Macaranga tanarius*. J. Nat. Prod..

[31] Lim T.Y., Lim Y.Y., Yule C.M. (2009). Evaluation of antioxidant, antibacterial and anti-tyrosinase activities of four *Macaranga* species. J. Food Chem..

[32] Raghukumar R., Vali L., Watson D., Fearnley J., Seidel V. (2010). Antimethicillin-resistant *Staphylococcus aureus* (MRSA) activity of ‘pacific propolis’ and isolated prenylflavanones. Phytother. Res..

[33] Matsunami K., Takamori I., Shinzato T., Aramoto M., Kondo K., Otsuka H., Takeda Y. (2006). Radical-scavenging activities of new megastigmane glucosides from *Macaranga tanarius* (L.) MULL.-ARG. Chem. Pharm. Bull..

